# Two-photon interference between independent atomic and quantum dot single-photon sources for hybrid quantum network

**DOI:** 10.1038/s41377-026-02399-y

**Published:** 2026-07-15

**Authors:** Kyu-Young Kim, Heewoo Kim, Dong Hyun Park, Jinhyuk Bae, Gyeongmin Ju, Suk In Park, Jin Dong Song, Je-Hyung Kim, Han Seb Moon

**Affiliations:** 1 Department of Physics, Ulsan National Institute of Science and Technology, Ulsan, Republic of Korea; 2 Department of Physics, Pusan National University, Busan, Republic of Korea; 3 Center for Opto-Electronic Materials and Devices Research, Korea Institute of Science and Technology, Seoul, Republic of Korea; 4 Quantum Sensors Research Center, Pusan National University, Busan, Republic of Korea

**Keywords:** Quantum optics, Quantum optics, Photonic devices

## Abstract

Hybrid quantum systems play a crucial role in advancing scalable and versatile quantum networks as they combine the strengths of different quantum platforms. An important challenge for the development of hybrid quantum networks lies in interfacing heterogeneous quantum nodes and distributing entanglement among them. Single photons emitted from these dissimilar quantum nodes typically show distinct spectral and temporal properties. Therefore, they necessitate emitters’ spectral modification and temporal synchronization, which introduce significant photon losses and require additional resources. In this work, we successfully generate indistinguishable photons from two distinct quantum systems: a warm atomic ensemble and a solid-state quantum dot. Remarkably, two-photon interference between dissimilar sources is achieved without additional spectral modification of single photons under continuous-wave operation and time-resolved coincidence detection, providing a practical route toward hybrid quantum nodes. A ^133^Cs atomic ensemble can efficiently generate heralded single photons at the wavelength of $917$ nm of the $6{{\rm{P}}}_{3/2}-6{{\rm{D}}}_{5/2}$ transition, while the single photons emitted from an InAs/GaAs quantum dot can be tuned to match the ^133^Cs transition wavelength. Our dense warm atomic ensemble and cavity-coupled quantum dot can efficiently generate bright and resonant single photons at detection rates approaching the MHz range, respectively. More importantly, these single photons exhibit inherent spectral similarity not only in the wavelength but also in the spectral linewidth, achieving a high spectral overlap of $0.88$. Such intrinsic compatibility between dissimilar quantum sources is essential to leverage the advantages of different quantum platforms, paving the way toward a large-scale and functional hybrid quantum network.

## Abstract (web summary)

Indistinguishable photons from atomic and quantum dot systems enable two-photon interference without spectral modification, providing a practical route toward scalable hybrid quantum networks.

## Graphical abstract

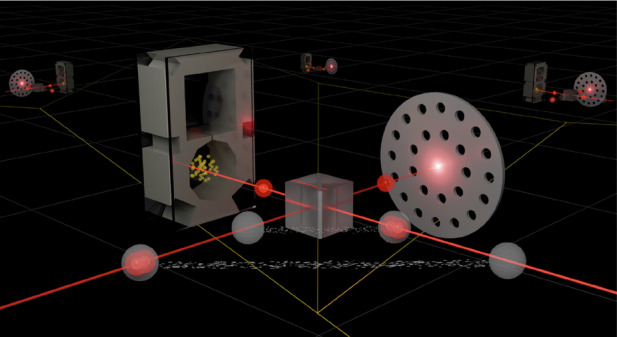

## Introduction

Quantum networks consist of quantum nodes and quantum channels, which are based on quantum memories and quantum light sources^1–3^. Implementing highly entangled photon states and entanglement swapping are also essential parts for correcting errors and extending the communication distance^4,5^. Achieving a source of coherent single photons that have narrow linewidth, high brightness, spectral uniformity, and compatibility with quantum memories is a critical requirement for a large-scale quantum network. A variety of quantum platforms, such as spontaneous parametric down-conversion (SPDC)^6,7^, atoms^3,8,9^, color centers^1,2,5^, and quantum dots (QDs)^10–13^, have been proposed and experimentally demonstrated for quantum key distributions, quantum teleportation, and entanglement distribution. Despite these advances, realizing a scalable and functional quantum network remains challenging due to the inherent limitations of each quantum platform, such as photon loss, limited coherence times, probabilistic operation, and integration difficulties.

Hybrid quantum architectures that combine different types of quantum light sources can address these challenges by leveraging the advantages of each platform. For example, QDs can serve as on-demand single photon sources with high brightness, high purity, and near Fourier-transform-limited linewidth^14–16^. These solid-state emitters are particularly suited for integration with nanophotonic structures for tailoring light-matter interactions^17,18^, as well as optical fibers^19^ or photonic-integrated chips^20,21^, enabling scalable miniaturization^22^. However, QDs suffer from spectral randomness and difficulties in storing photons, which limit long-distance interactions between remote QDs. In contrast, atomic systems with their naturally identical energy levels ensure uniform interactions across quantum nodes^3,8,23^ and offer reliable frequency standards within a quantum network. This will allow the distribution of tasks in a quantum network such that QDs handle high-rate photon generation for fast transmission, while atoms manage storage and synchronization of distant QD emissions^24–26^.

To realize quantum interference and to physically implement such a modular quantum architecture, achieving high identity of emissions from each source in both central frequency and physical modes, such as spectral and temporal profiles, is essential. Previously, two-photon interference (TPI) between dissimilar sources has been demonstrated from various platforms, including sunlight (thermal light)-QD^27,28^, laser (coherent light)-QD^29,30^, and SPDC (heralded single photons)-QD^31,32^. However, these approaches require spectral modifications of single photons to compensate for large mismatches in spectral linewidth between the sources. Furthermore, pulse-mode operation across the experimental system, from sample excitation to detection and post-selection, was used to define temporal modes and to identify TPI events from the dissimilar sources. Although these results highlight the feasibility of TPI from hybrid quantum systems, their complexity and inefficiency of the system still present considerations for practical implementation.

Warm atomic ensembles are ideal platforms compatible with QDs. For example, the $780$ nm transition of Rb atoms^33^ and the $917$ nm transition of Cs atoms^34^ spectrally align with GaAs/AlGaAs QDs^15^ and InAs/GaAs QDs^18,35^, respectively. Moreover, warm atomic ensembles exhibit a spectral linewidth ranging from hundreds of MHz to several GHz^36,37^, comparable to that of QDs. These spectral linewidths and the natural wavelength compatibility make warm vapor cells attractive building blocks for hybrid atom–QD interfaces. In particular, atomic-vapor-cell-based quantum light sources have been widely investigated as compact and operationally simple platforms for generating narrowband quantum light^33,34,38^. Notably, bright and robust spontaneous four-wave mixing (SFWM) photon pairs generated from warm atomic vapor cells have successfully served as crucial resources for experimental realization in quantum memory, quantum repeaters, and long-distance quantum networks^3,23,39,40^. Despite the strong optical compatibility between warm atomic ensembles and QDs, previous studies^25,26,41^ have been limited to using warm atomic ensembles as dispersive media for photons emitted from QDs. Direct TPI between single photons from these two quantum sources has remained unexplored.

Here, we demonstrate TPI between single photons emitted from a single QD and a warm atomic ensemble. Specifically, ^133^Cs atoms generate heralded single photons at a well-defined $917$ nm, which lies within the emission range of InAs/GaAs QDs. Employing fine spectral tuning of the QD emission enables spectral matching between two quantum sources with a spectral overlap of around $0.88$. Utilizing the thin vapor cell and nano-cavity integration, both quantum systems experimentally produce single photons efficiently at detection rates approaching the MHz range. As a result, two independent quantum systems efficiently demonstrate TPI without tight spectral modification of single photons under continuous operation. Our result provides fundamental insight into how quantum coherence and indistinguishability can be preserved across different physical platforms. Furthermore, our approach establishes a critical step toward interfacing two different quantum systems through optical channels and paves the way for scalable hybrid quantum networks that support remote entanglement generation and quantum teleportation between dissimilar quantum platforms^42–44^.

## Results

TPI between dissimilar sources requires frequency matching as well as similar coherence times so that the emitted photons are indistinguishable. Before we conduct TPI between photons from a warm atomic ensemble and a single QD, we characterize the optical properties of each single-photon source.

### Heralded single-photon generation via SFWM in a warm ^133^Cs ensemble

**Fig. 1:**
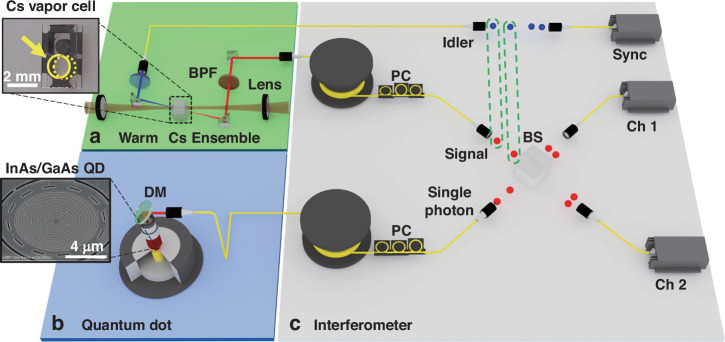
Experimental setup of the TPI between two different quantum emitters. **a** Generation of heralded single photons from a warm ^133^Cs ensemble (Green section). The inset shows an image of a cell filled with a warm ^133^Cs atomic ensemble. **b** Deterministic generation of single photons from a cavity-coupled QD, cooled by a He-closed cryostat (Blue section). The inset shows a scanning electron microscopy image of the fabricated hole-based circular Bragg grating cavity. **c** Setup for TPI where a signal-idler photon-pair from an atomic cell and a single photon from a QD interfere (Gray section). BPF band-pass filter, DM dichroic mirror, PC polarization controller, BS beamsplitter

**Fig. 2:**
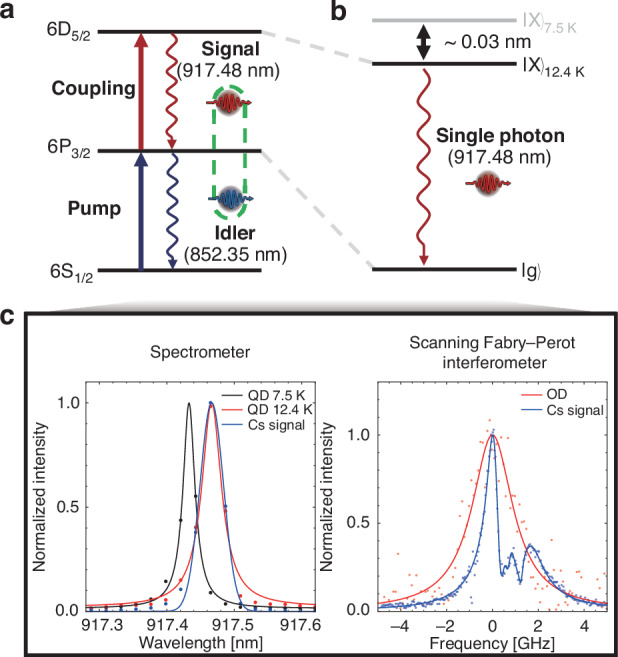
Spectral characteristics of two distinct quantum systems. **a** Description of the ladder-type energy level of warm ^133^Cs atoms. Red and blue solid arrows indicate the counter-propagating coupling and pump CW lasers. Red and blue curved arrows indicate the correlated signal and idler photons via the SFWM process. **b** Energy level of a single QD before and after thermal tuning. After thermal tuning, the transition energy becomes resonant with the signal photon of ^133^Cs atoms at 12.4 K. **c** Comparison of spectra of the photons from the warm ^133^Cs ensemble and the QD by a spectrometer (left) and high-resolution spectra by a scanning Fabry-Perot interferometer (right). Black and red solid lines represent single photons from the cavity-coupled QD at $7.5$ and $12.4$ K, respectively. Blue solid lines are the spectra of the signal photons from the warm ^133^Cs ensemble

A warm ^133^Cs ensemble generates heralded single photons via the SFWM process from its $6{{\rm{S}}}_{1/2}-6{{\rm{P}}}_{3/2}-6{{\rm{D}}}_{5/2}$ transition^34^. Figure 1a shows an optical setup of heralded single-photon generation using a thin, mm-scale warm ^133^Cs vapor cell^34^ (Inset of Fig. 1a). Two counter-propagating continuous-wave (CW) lasers, called pump and coupling, were sent through a warm ^133^Cs ensemble (Fig. 1a), generating correlated signal (917.48 nm) and idler (852.35 nm) photon pairs (Fig. 2a). The signal photon count rate was controlled by varying the pump laser power and was tuned between $0.11\pm 0.04$ MHz and $0.88\pm 0.15$ MHz during the experiment. In photon-pair generation experiments using an atomic vapor cell, reabsorption effects set an optimal range for the optical depth^37,45^. Employing a thin vapor cell improves the spatial overlap between the pump and the generated modes, thereby suppressing reabsorption losses. As a result, the source exhibits higher brightness and an improved signal-to-noise ratio at a higher optical depth^34^. Under these optimized conditions, the heralding efficiency was significantly improved, reaching as high as 22%. The detailed experimental condition is explained in Methods. The spectrum of the signal photons is shown as a blue solid line in Fig. 2c (left), but the measured spectral linewidth of the heralded single photon from the warm ^133^Cs ensemble is limited by the spectrometer’s resolution (7 GHz). By using a scanning Fabry-Perot interferometer, as shown in the blue solid line in Fig. 2c (right), we directly measured the spectrum of heralded single photons, corresponding to the biphoton spectral wavefunction of our photon pairs.

### Single-photon generation from the quantum dot

Self-assembled InAs/GaAs QDs are among the most promising platforms for generating high-performance single photons, and their emissions lie around the $6{{\rm{P}}}_{3/2}-6{{\rm{D}}}_{5/2}$ transition of ^133^Cs. However, unlike atomic transitions, the inhomogeneous nature of QDs leads to their emission over a wide spectral distribution. To achieve spectrally resonant emission from a QD, we fabricated a nanophotonic cavity designed at $917$ nm (see Methods). The hole-based circular Bragg grating low-*Q* cavity (Inset of Fig. 1b) can be coupled with QDs over a broad spectral range, two orders of magnitude broader than single QDs’ linewidth^18,19,22^. Finite-difference time-domain simulation shows that single photons emitted from the cavity-coupled QDs exhibit high vertical directionality, improving coupling efficiency with a single-mode optical fiber (${\rm{NA}}=0.13$) up to $27 \%$.

We first found a cavity-coupled bright QD near the atomic transition of $917.48$ nm. The QD was cooled to cryogenic temperature and optically excited using a CW laser at $780$ nm (Fig. 1b). The experimental single photon count rate reaches $0.44\pm 0.04$ MHz. At the lowest temperature of 7.5 K, the QD exhibits a spectral detuning of 0.03 nm relative to the heralded signal photons, along with a spectrometer-limited linewidth (Fig. 2c (left)). To compensate for the small spectral mismatch between the QD emission and the heralded signal photons from the warm ^133^Cs ensemble, we red-shifted the QD emission by increasing the sample temperature, achieving resonance at $12.4$ K, as shown in Fig. 2b, c (left). We note that increasing the sample temperature also leads to linewidth broadening due to enhanced phonon interaction and thermal activation of carriers^46^, as shown in Fig. 2c.

### Indistinguishability between photons from warm ^133^Cs ensemble and QD

Photon indistinguishability,$\,{I}_{{\rm{id}}}$, is the degree of similarity between the single photons from the QD and heralded signal photons from the warm ^133^Cs ensemble in terms of wavelength, polarization, spatial-temporal modes, and other degrees of freedom, and is a continuous parameter ranging from 0 to 1, corresponding to perfectly distinguishable and indistinguishable photons, respectively. When the spectral overlap, $A$, is given as follows^47^:

1 $$A\left({S}_{{\rm{QD}}},{S}_{{\rm{s}}}\right)=\frac{{\left|\int \sqrt{{S}_{{\rm{QD}}}\left(\omega \right)\cdot {S}_{{\rm{s}}}\left(\omega \right)}d\omega \right|}^{2}}{\int {S}_{{\rm{QD}}}\left(\omega \right)d\omega \cdot \int {S}_{{\rm{s}}}\left(\omega \right)d\omega }$$

where ${S}_{{\rm{QD}}}$ is the QD emission spectrum and ${S}_{{\rm{s}}}$ is the spectrum of signal photons heralded by coincidence with the idler photons, the photon indistinguishability, ${I}_{{\rm{id}}}$, is equivalent to $A$ provided that the photons are indistinguishable in all other degrees of freedom. To quantify the spectral similarity between two sources beyond the spectrometer’s resolution, we compare their high-resolution spectra measured using the scanning Fabry-Perot interferometer with a transmission window of less than $55$ MHz under spectrally resonant conditions. The red solid line in Fig. 2c (right) represents a Lorentzian fit (${S}_{{\rm{QD}}}$) to the QD spectrum at $12.4$ K, with a full width at half maximum (FWHM) of $2.17\pm 0.17$ GHz. The heralded signal photon spectrum (${S}_{{\rm{s}}}$, blue solid line in Fig. 2c (right)) from the warm ^133^Cs ensemble exhibits a more complex profile due to both spectral deformation caused by hyperfine structures of $6{{\rm{P}}}_{3/2}$ and $6{{\rm{D}}}_{5/2}$ levels and photon reabsorption^48^. From the measured ${S}_{{\rm{QD}}}$ and ${S}_{{\rm{s}}}$, we determine a spectral overlap of *A* = 0.88 ± 0.01 without spectral modification of either source. The uncertainty of $A$ was estimated by error propagation from the fitted spectral parameters of ${S}_{{\rm{QD}}}$ and ${S}_{{\rm{s}}}$. Therefore, the spectral overlap imposes an upper bound of $0.88$ on the photon indistinguishability in our system.

This high degree of spectral identity between the two dissimilar quantum light sources is sufficient to enable high-visibility TPI, as required for non-classical correlation measurement^49,50^. In previous studies^27,28,31,32^, etalon filters with narrow transmission bandwidths were inevitable due to the large mismatch in the spectral profiles between the sources. This causes a significant loss in their emission and lowers the rate of TPI. In contrast, the warm ^133^Cs ensemble and the single QD experimentally generate single photons with high brightness of near MHz single photon count rates and high spectral similarity. This intrinsic compatibility ensures TPI without photon losses, representing a key advantage of a hybrid quantum architecture based on a warm atomic ensemble and semiconductor QDs.

### Photon correlations and coherence times

**Fig. 3:**
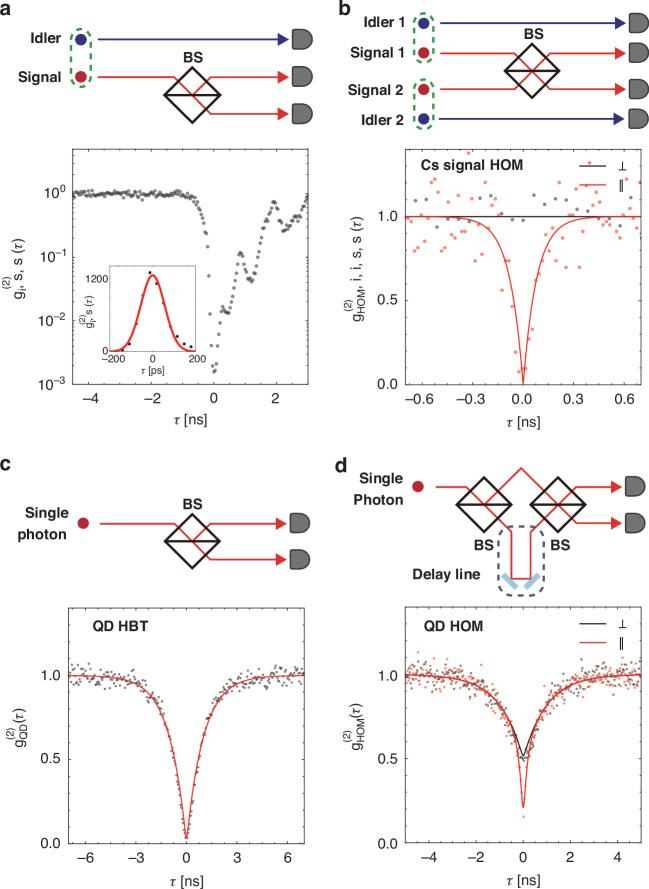
Characterizations of single photons from the warm ^133^Cs ensemble and the QD with their coherence properties. **a** Conditional HBT measurement of the signal photons from the warm ^133^Cs ensemble by using a three-fold coincidence setup. The inset is the cross-correlation curve between the signal and idler photons, exhibiting strong coincidence counts with the Gaussian FWHM of $128\pm 4$ ps. **b** HOM interference curves for the signal photons using a four-fold coincidence setup. The black and red lines represent interference patterns for orthogonal (distinguishable) and parallel (indistinguishable) polarization of two input signal photons, respectively. **c** HBT measurement of the single photon from the cavity-coupled QD at $12.4$ K. **d** HOM interference measurement of single photons from the QD. The black (red) line represents the interference pattern for the orthogonal (parallel) polarization case. Schematics above the graphs represent brief experimental schemes for correlation measurements. A 4 m delay line was inserted in one arm to interfere with two temporally separated single photons. All figures are plotted with a time bin width of $40$ ps

We then perform single-photon characterization of each source by correlation measurements. First, we characterized the cross-correlation, ${{\rm{g}}}_{{\rm{i}},{\rm{s}}}^{\left(2\right)}\left(\tau \right)$, between the signal and idler photons from the warm ^133^Cs ensemble. (Supplementary Note 1) Inset of Fig. 3a shows that the measured ${{\rm{g}}}_{{\rm{i}},{\rm{s}}}^{\left(2\right)}\left(\tau \right)$ presents strong bunching peak, exceeding ${{\rm{g}}}_{{\rm{i}},{\rm{s}}}^{\left(2\right)}\left(0\right)=1245\pm 4$ at zero-time delay for a single-count rate of 0.88 MHz. Under our normalization, the coincidence-to-accidental ratio (CAR) is given by ${{\rm{g}}}_{{\rm{i}},{\rm{s}}}^{\left(2\right)}\left(0\right)={\rm{CAR}}+1$ corresponding to ${\rm{CAR}}=\mathrm{1,244}\pm 4$. The accidental coincidence level was estimated from the coincidence histogram at infinite time delays and normalized to unity. The result indicates strong temporal correlations between signal and idler photons. From the fitting with a Gaussian function (red solid line in Fig. 3a), we determine the heralded signal photons effectively serve as single photons within heralding time window of $128\pm 4$ ps FWHM. Owing to the use of a thin vapor cell, the heralding efficiency is estimated to be approximately $18$% within the experimental heralding time window of $320$ ps at individual signal and idler photon count rates of $0.88$ MHz. The achieved heralding efficiency is higher than that in previous studies^34,45^.

Then, using the idler photons as heralding triggers within the experimental heralding time window of $80$ ps, we measured the three-fold coincidence count of the heralded signal photons. (Supplementary Note 1 and 2) To reveal the single photon nature of the heralded signal photons, we normalize the three-fold coincidence result by the measured cross-correlation between idler and signal photons (Inset in Fig. 3a) and get the normalized conditional second-order correlation (${{\rm{g}}}_{{\rm{i}},{\rm{s}},{\rm{s}}}^{\left(2\right)}\left(\tau \right)$) of heralded signal photons from the warm ^133^Cs ensemble (Fig. 3a)^33^. The antibunching feature is ${{\rm{g}}}_{{\rm{i}},{\rm{s}},{\rm{s}}}^{\left(2\right)}\left(0\right)=0.002$, representing the high single-photon purity of the heralded signal photons. The oscillatory features observed in ${{\rm{g}}}_{{\rm{i}},{\rm{s}},{\rm{s}}}^{\left(2\right)}\left(\tau \right)$ is closely related to the complex frequency spectrum of the heralded signal photon in Fig. 2c (right), which arises from reabsorption of idler photons^38,48^. The modified spectrum of the heralded signal photons leads to quantum beating between the hyperfine levels of 6P_3/2_ and 6D_5/2_ states in ^133^Cs atoms.

To characterize the coherence property of the heralded signal photons, we perform TPI between two heralded signal photons generated in two phase-matched directions at ±2° relative to the pump–coupling beam axis from the same ^133^Cs vapor cell, using the fourth-order coincidence of two idler photons as heralding triggers and two interfering signal photons. (Supplementary Note 1) To control the indistinguishability of the heralded signal photon, we adjusted the polarizations of the signal photons before the photons entered the BS. With an $80$ ps heralding window applied to the signal-idler photon pairs, we observed a raw Hong-Ou-Mandel (HOM) dip of ${{\rm{g}}}_{{\rm{HOM}},{\rm{i}},{\rm{i}},{\rm{s}},{\rm{s}}}^{\left(2\right)}\left(0\right)=0.10$ when input photons were parallel, while the antibunching dip disappeared when photons were orthogonally polarized as shown in Fig. 3b. From a theoretical fit to Fig. 3b, we obtained a HOM visibility of $1.00\pm 0.11$. Given the coherence time ($161\pm 28$ ps) of signal photons from the warm ^133^Cs ensemble, the large HOM interference pattern contrast shows the high indistinguishability of the heralded single photons from the warm ^133^Cs ensemble.

Next, we characterize the single-photon properties of the single QD. In contrast to the heralded single photons from the warm ^133^Cs ensemble, the single photons from the single QD do not require a heralding process. It shows antibunching behavior via a direct Hanbury Brown and Twiss (HBT) experiment. Figure 3c shows the measured second-order correlation curve of the single QD at 12.4 K. To account for a temporal resolution of the system, we convolved the fitting function,

2 $${{\rm{g}}}_{{\rm{QD}}}^{\left(2\right)}\left(\tau \right)=1-\left(1-{{\rm{g}}}_{{\rm{QD}}}^{\left(2\right)}\left(0\right)\right)\exp \left[-\left|\tau \right|/{\tau }_{{\rm{QD}}}\right]$$

with a convolution of timing jitter ($104$ ps) of two superconducting nanowire single-photon detectors (SNSPDs) and a time-correlated single photon counting (TCSPC), where ${\tau }_{{\rm{QD}}}=1.01\pm 0.02$ ns is the lifetime of a QD. As a result of the low-*Q* cavity coupling, the single-photon count rate reached $0.44$ MHz, and the pronounced antibunching profile with ${{\rm{g}}}_{{\rm{QD}}}^{\left(2\right)}\left(0\right)=0.01\pm 0.01$ confirms the generation of high-purity single photons from the single QD. (Supplementary Note 3) We subsequently performed HOM interference on the single photons from the single QD to assess their indistinguishability and coherence time. (Supplementary Note 1) Temporally separated single photons were interfered with at a 50:50 BS within an asymmetric Mach-Zehnder interferometer with an optical delay of 4 m (=19.6 ns) in one of the interferometer arms. Figure 3d displays HOM results for the cases: two photons with orthogonal (black, distinguishable) and parallel (red, indistinguishable) polarizations after convolving the temporal resolution of our system. A HOM interference dip below $0.5$ appears when the input photons are indistinguishable (${{\rm{g}}}_{{\rm{HOM}},\,\parallel }^{\left(2\right)}\left(0\right) < 0.5$). From the calculation of ${V}_{{\rm{QD}}}\left(\tau \right)=\left({{\rm{g}}}_{{\rm{HOM}},\,\perp }^{\left(2\right)}\left(\tau \right)-{{\rm{g}}}_{{\rm{HOM}},\,\parallel }^{\left(2\right)}\left(\tau \right)\right)/{{\rm{g}}}_{{\rm{HOM}},\,\perp }^{\left(2\right)}\left(\tau \right)$, we obtained uncorrected single-photon visibility of ${V}_{{\rm{QD}}}\left(0\right)=0.62\pm 0.07$ at $12.4$ K. After deconvolving the temporal resolution of our system, we finally obtained ${V}_{{\rm{QD}}}\left(0\right)=1.00\pm 0.10$, representing a high degree of single-photon indistinguishability with a coherence time of ${\tau }_{{\rm{QD}},{\rm{c}}}=129\pm 18$ ps (Supplementary Note 3). Through these characterizations, we compared two different types of single-photon sources and confirmed physical similarities.

### Two-photon interference between heterogeneous single photon sources

TPI between single photons from the warm ^133^Cs ensemble and the QD platforms can be characterized via the three-fold coincidence measurement scheme illustrated in Fig. 1c. Experimentally, we directly obtain three-fold coincidence counts, ${C}_{{\rm{i}},{\rm{s}},{\rm{QD}}}\left(\tau \right)$, from detection at the two output ports of the BS, which mixes the heralded signal photons from the warm ^133^Cs ensemble and single photons from the QD. These coincidence events are conditioned on the detection of the idler photons within a predefined heralding time window. (Supplementary Note 1) As discussed above (Fig. 3a), to reveal the single photon nature of the heralded single photons, we normalized the three-fold coincidence by the measured cross-correlation between signal and idler photons. Similarly, the conditional second-order correlation for TPI,$\,{{\rm{g}}}_{{\rm{i}},{\rm{s}},{\rm{QD}}}^{\left(2\right)}\left(\tau \right)$, requires appropriate normalization. ${{\rm{g}}}_{{\rm{i}},{\rm{s}},{\rm{QD}}}^{\left(2\right)}\left(\tau \right)$ can be obtained by normalizing ${C}_{{\rm{i}},{\rm{s}},{\rm{QD}}}\left(\tau \right)$ by the cross-correlation between the idler photon detection and one output of the BS (Supplementary Note 4), which can be expressed as follows^33^:

3 $${{\rm{g}}}_{{\rm{i}},{\rm{s}},{\rm{QD}}}^{\left(2\right)}\left(\tau \right)=\frac{{N}_{{\rm{i}}}}{{N}_{{\rm{i}},1\left(2\right)}}\cdot \frac{{C}_{{\rm{i}},{\rm{s}},{\rm{QD}}}\left(\tau \right)}{{C}_{{\rm{i}},2\left(1\right)}\left(\tau \right)}$$

where ${N}_{{\rm{i}}}$ and ${N}_{{\rm{i}},1\left(2\right)}$ are the count rates of idler photons and the coincidences between heralding idler photons and the first (second) output port of the BS at zero-time delay, respectively, and ${C}_{{\rm{i}},1\left(2\right)}\left(\tau \right)$ is the two-fold coincidence between heralding idler photons and the first (second) output port of the BS. Since ${N}_{{\rm{i}}}$, $\,{N}_{{\rm{i}},1\left(2\right)}$, and ${C}_{{\rm{i}},1(2)}\left(\tau \right)$ are independent of the indistinguishability of interfering photons (Supplementary Note 5), the time-dependent HOM visibility, ${V}_{{\rm{i}},{\rm{s}},{\rm{QD}}}\left(\tau \right)$, can be expressed as:

4 $${V}_{{\rm{i}},{\rm{s}},{\rm{QD}}}\left(\tau \right)=\frac{{{\rm{g}}}_{{\rm{i}},{\rm{s}},{\rm{QD}},\perp }^{\left(2\right)}\left(\tau \right)-{{\rm{g}}}_{{\rm{i}},{\rm{s}},{\rm{QD}},\parallel }^{\left(2\right)}\left(\tau \right)}{{{\rm{g}}}_{{\rm{i}},{\rm{s}},{\rm{QD}},\perp }^{\left(2\right)}\left(\tau \right)}=\frac{{C}_{{\rm{i}},{\rm{s}},{\rm{QD}},\perp }\left(\tau \right)-{C}_{{\rm{i}},{\rm{s}},{\rm{QD}},\parallel }\left(\tau \right)}{{C}_{{\rm{i}},{\rm{s}},{\rm{QD}},\perp }\left(\tau \right)}$$

where ${{\rm{g}}}_{{\rm{i}},{\rm{s}},{\rm{QD}},\perp \left(\parallel \right)}^{\left(2\right)}\left(\tau \right)$ and ${C}_{{\rm{i}},{\rm{s}},{\rm{QD}},\perp \left(\parallel \right)}\left(\tau \right)$ are the conditional second-order correlation and the three-fold coincidence for distinguishable (indistinguishable) photon inputs. ${C}_{{\rm{i}},{\rm{s}},{\rm{QD}}}\left(\tau \right)$ can be directly measured without any time-dependent normalization process in contrast to ${{\rm{g}}}_{{\rm{i}},{\rm{s}},{\rm{QD}}}^{\left(2\right)}\left(\tau \right)$. Therefore, we use the three-fold coincidence to calculate the TPI visibility.

**Fig. 4:**
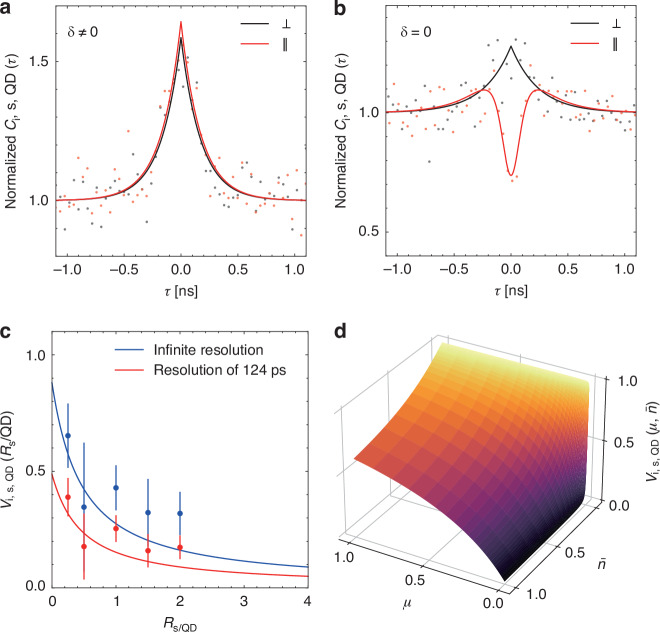
TPI between the single photons and the heralded signal photons. **a** TPI measurement when the QD is spectrally detuned ($\delta \ne 0$) from the warm ^133^Cs ensemble transition. Three-fold coincidence results are normalized to the accidental coincidence level at large time delay. The black and red data represent distinguishable (orthogonal) and indistinguishable (parallel) in the polarization basis, respectively. The corresponding fitted bunching amplitudes are ${C}_{{\rm{i}},{\rm{s}},{\rm{QD}}}^{{\rm{dis}}}\left(0\right)=1.59\pm 0.05$ and ${C}_{{\rm{i}},{\rm{s}},{\rm{QD}}}^{{\rm{indis}}}\left(0\right)=1.64\pm 0.03$. **b** TPI measurements for resonant ($\delta =0$), which is normalized to the accidental coincidence level obtained at large time delays, between the two single photons from the QDs and the warm ^133^Cs ensemble when ${R}_{{\rm{s}}/{\rm{QD}}}=0.25$. The normalized three-fold coincidence amplitudes are ${C}_{{\rm{i}},{\rm{s}},{\rm{QD}}}^{{\rm{dis}}}\left(0\right)=1.28\pm 0.04$ and ${C}_{{\rm{i}},{\rm{s}},{\rm{QD}}}^{{\rm{indis}}}\left(0\right)=0.56\pm 0.07$. **c** Experimentally measured (points) and theoretically calculated (solid lines) TPI visibilities with varying ${R}_{{\rm{s}}/{\rm{QD}}}$. The red and blue data represent measured and calculated results with and without accounting for the system temporal resolution. **d** Analytical model for the TPI visibility (${V}_{{\rm{i}},{\rm{s}},{\rm{QD}}}\left(0\right)$) as a function of mean photon number ($\bar{n}$) of unheralded signal photons and the system efficiency ($\mu$) of the QD under the ideal condition of the perfect indistinguishability (${I}_{{\rm{id}}}=1$). All figures are plotted with a time bin width of $40$ ps

Finally, we demonstrate TPI between single photons from the QD and heralded signal photons from the warm ^133^Cs ensemble under continuous excitation. To implement the three-fold coincidence scheme discussed above, the QD and signal photons were directed into the two input ports of the BS, while the correlated idler photons provided the heralding trigger. To investigate both indistinguishable and distinguishable cases, we adjusted the relative polarization between the input photons. The output photons from the BS were then detected by two SNSPDs, labeled Ch1 and Ch2, while the idler photons were separately recorded by the Sync channel. By post-selecting Ch1-Ch2 detection events within 80 ps after the idler detection, three-fold coincidence, ${C}_{{\rm{i}},{\rm{s}},{\rm{QD}}}\left(\tau \right)$, was obtained. For comparison, we first performed TPIs when the QD and the warm ^133^Cs ensemble are spectrally detuned ($\delta =0.03$ nm). In Fig. 4a, the results show that there is no visible change in their coincidence events between orthogonal and parallel polarization cases, which is expected since they are spectrally distinguishable. Then, we performed TPI for the case of two resonant sources after spectral tuning of the QD. The TPI suppresses coincidence events at zero-time delay when two single photons are parallel, and we observe a large interference contrast from the case when two photons are orthogonal (Fig. 4b). This comparison confirms that spectral identity is essential for TPI between photons from two different quantum light sources.

To quantify the TPI, we fit the ${C}_{{\rm{i}},{\rm{s}},{\rm{QD}}}\left(\tau \right)$, normalized to the accidental coincidence level, with the following equation that describes bunching and antibunching profile in the three-fold coincidence curves:

5 $${C}_{{\rm{i}},{\rm{s}},{\rm{QD}}}\left(\tau \right)=\left(1+\left({C}_{{\rm{i}},{\rm{s}},{\rm{QD}},\perp }\left(0\right)-1\right)\exp \left[-2\left|\tau \right|/{\tau }_{{\rm{hs}}}\right]\right)\times \left(1-{V}_{{\rm{i}},{\rm{s}},{\rm{QD}}}\left(0\right)\exp \left[-2\left|\tau \right|/{\tau }_{{\rm{i}},{\rm{s}},{\rm{QD}}}\right]\right)$$

Here, ${\tau }_{{\rm{hs}}}$ denotes the coherence time of heralded signal photons, corresponding to the temporal wavepacket of signal photons with heralding process. It is obtained from the three-fold coincidence measurement of heralded signal photons. (Supplementary Note 2) The parameter ${\tau }_{{\rm{i}},{\rm{s}},{\rm{QD}}}$ is the TPI coherence time, corresponding to the temporal overlap between the heralded signal photons from the warm Cs ensemble and single photons from the QD. ${V}_{{\rm{i}},{\rm{s}},{\rm{QD}}}\left(0\right)$ is the TPI visibility between interfered signal photons from the warm ^133^Cs ensemble and single photons from the QD^51^. For the perfectly distinguishable limit, ${V}_{{\rm{i}},{\rm{s}},{\rm{QD}}}\left(0\right)=0$, and the ${C}_{{\rm{i}},{\rm{s}},{\rm{QD}}}\left(\tau \right)$ is dominated by the three-fold coincidence characteristic of idler and signal photons, leading to a bunching profile in the curve within the coherence time (${\tau }_{{\rm{hs}}}$) among heralded signal photons. For indistinguishable photons, ${V}_{{\rm{i}},{\rm{s}},{\rm{QD}}}\left(0\right)$ takes values between 0 and 1, and the antibunching profile emerges as a result of TPI within the TPI coherence time (${\tau }_{{\rm{i}},{\rm{s}},{\rm{QD}}}$) of the heralded signal photons from the warm Cs ensemble and single photons from the QD.

From the calculation of Eq. (4), we obtained a raw TPI visibility of ${V}_{{\rm{i}},{\rm{s}},{\rm{QD}}}\left(0\right)=0.41$ with the three-fold coincidence rate of 1.07 ± 0.14 Hz. This measured visibility is limited by the finite system temporal resolution of 124 ps, which is comparable to the coherence time (*τ*_i,s,QD_) of 119 ps of single photons. To account for the finite system temporal resolution, we convolved the time-dependent TPI visibility curve with our system’s temporal response function and corrected the TPI visibilities. (Supplementary Note 3) From the deconvolution process, the corrected visibility rises to ${V}_{{\rm{i}},{\rm{s}},{\rm{QD}}}\left(0\right)=0.65\pm 0.14$.

The imbalance in detected single-photon rates between the atomic and QD channels is another major factor that limits the achievable visibility, even after temporal deconvolution. To investigate the influence of the imbalance in detected single-photon rates, we define ${R}_{{\rm{s}}/{\rm{QD}}}$ as the ratio of the detected single-photon count rate of unheralded signal photons from the warm ^133^Cs ensemble to that of single photons from the QD. We investigate TPI visibility according to ${R}_{{\rm{s}}/{\rm{QD}}}$ by increasing the count rate of unheralded signal photons from the warm ^133^Cs ensemble (from $0.11$ MHz to $0.88$ MHz) while fixing the count rate of single photons from the QD ($0.44$ MHz). Figure 4c shows that the measured visibilities decrease with ${R}_{{\rm{s}}/{\rm{QD}}}$.

To analyze how ${R}_{{\rm{s}}/{\rm{QD}}}$ affects TPI visibility, we developed a theoretical model under realistic lossy conditions and performed numerical calculations. Analyzing TPI between the single photons from the QD and heralded signal photons from the warm ^133^Cs ensemble involves the three-fold coincidence among Ch1 and Ch2 (interfered photons) and Sync (the idler photons), defined as:

6 $${C}_{{\rm{i}},{\rm{s}},{\rm{QD}}}\left(0\right)=\left\langle {\hat{a}}_{{\rm{i}}}^{\dagger }{\hat{a}}_{{\rm{Ch}}1}^{\dagger }{\hat{a}}_{{\rm{Ch}}2}^{\dagger }{\hat{a}}_{{\rm{Ch}}2}{\hat{a}}_{{\rm{Ch}}1}{\hat{a}}_{{\rm{i}}}\right\rangle$$

where ${\hat{a}}_{{\rm{Ch}}1}^{\dagger }$, ${\hat{a}}_{{\rm{Ch}}2}^{\dagger }$, and ${\hat{a}}_{{\rm{i}}}^{\dagger }$ are creation operators for photons detected at Ch1, Ch2, and Sync, respectively. Following reference^52^, the three-fold coincidence can be expressed as

7 $${C}_{{\rm{i}},{\rm{s}},{\rm{QD}}}\left(0\right)=\left[\left\langle {\hat{a}}_{{\rm{i}}}^{\dagger }{\hat{a}}_{{\rm{QD}}}^{\dagger }{\hat{a}}_{{\rm{QD}}}^{\dagger }{\hat{a}}_{{\rm{QD}}}{\hat{a}}_{{\rm{QD}}}{\hat{a}}_{{\rm{i}}}\right\rangle +\left\langle {\hat{a}}_{{\rm{i}}}^{\dagger }{\hat{a}}_{{\rm{s}}}^{\dagger }{\hat{a}}_{{\rm{s}}}^{\dagger }{\hat{a}}_{{\rm{s}}}{\hat{a}}_{{\rm{s}}}{\hat{a}}_{{\rm{i}}}\right\rangle +2\left\langle {\hat{a}}_{{\rm{QD}}}^{\dagger }{\hat{a}}_{{\rm{QD}}}\right\rangle \left\langle {\hat{a}}_{{\rm{i}}}^{\dagger }{\hat{a}}_{{\rm{s}}}^{\dagger }{\hat{a}}_{{\rm{s}}}{\hat{a}}_{{\rm{i}}}\right\rangle \left(1-{I}_{{\rm{id}}}\right)\right]$$

where ${\hat{a}}_{{\rm{QD}}}^{\dagger }$ and ${\hat{a}}_{{\rm{s}}}^{\dagger }$ are creation operators for the single photons from the QD and the signal photons from the warm ^133^Cs ensemble before entering the BS, and ${I}_{{\rm{id}}}$ is the photon indistinguishability. (Supplementary Note 5) According to the definition of the TPI visibility, which is relative contrast between distinguishable and indistinguishable cases, the visibility can be expressed as follow:

8 $${V}_{{\rm{i}},{\rm{s}},{\rm{QD}}}\left(0\right)=\frac{2\left\langle {\hat{a}}_{{\rm{QD}}}^{\dagger }{\hat{a}}_{{\rm{QD}}}\right\rangle \left\langle {\hat{a}}_{{\rm{i}}}^{\dagger }{\hat{a}}_{{\rm{s}}}^{\dagger }{\hat{a}}_{{\rm{s}}}{\hat{a}}_{{\rm{i}}}\right\rangle }{\left\langle {\hat{a}}_{{\rm{i}}}^{\dagger }{\hat{a}}_{{\rm{QD}}}^{\dagger }{\hat{a}}_{{\rm{QD}}}^{\dagger }{\hat{a}}_{{\rm{QD}}}{\hat{a}}_{{\rm{QD}}}{\hat{a}}_{{\rm{i}}}\right\rangle +\left\langle {\hat{a}}_{{\rm{i}}}^{\dagger }{\hat{a}}_{{\rm{s}}}^{\dagger }{\hat{a}}_{{\rm{s}}}^{\dagger }{\hat{a}}_{{\rm{s}}}{\hat{a}}_{{\rm{s}}}{\hat{a}}_{{\rm{i}}}\right\rangle +2\left\langle {\hat{a}}_{{\rm{QD}}}^{\dagger }{\hat{a}}_{{\rm{QD}}}\right\rangle \left\langle {\hat{a}}_{{\rm{i}}}^{\dagger }{\hat{a}}_{{\rm{s}}}^{\dagger }{\hat{a}}_{{\rm{s}}}{\hat{a}}_{{\rm{i}}}\right\rangle }{I}_{{\rm{id}}}$$

Taking into account our experimental system efficiencies of ${\eta }_{{\rm{s}}}=0.37$ and ${\eta }_{{\rm{i}}}=0.57$ for the signal and idler photons (Supplementary Note 6) and the photon indistinguishability of ${I}_{{\rm{id}}}=0.88$, obtained from the measured spectral overlap, we numerically calculated the TPI visibility with ${R}_{{\rm{s}}/{\rm{QD}}}$. The red curve in Fig. 4c represents the expected TPI visibility for the coherence time (*τ*_i,s,QD_) of 119 ps, obtained by convolving the ideal time-dependent TPI visibility function with the finite temporal resolution of 124 ps. (Supplementary Note 3) The blue curve plots the theoretical model, assuming infinitesimal temporal resolution, which enhances the TPI visibility. All experimental data closely follow the theoretical predictions with the system efficiency for single photons from the QD of $\mu =0.019$. (Supplementary Note 6)

Figure 4d shows the calculated TPI visibility as a function of system efficiency for the single photons generated by the QD ($\mu$) and a mean photon number of unheralded signal photons from the ^133^Cs ensemble ($\bar{n}$) at the ideal condition of the perfect indistinguishability (${I}_{{\rm{id}}}=1$). At the high mean photon number of unheralded signal photons, the multi-photon component becomes non-negligible. In Eq. (8), this effect manifests as an increase in the term $\left\langle {\hat{a}}_{{\rm{i}}}^{\dagger }{\hat{a}}_{{\rm{s}}}^{\dagger }{\hat{a}}_{{\rm{s}}}^{\dagger }{\hat{a}}_{{\rm{s}}}{\hat{a}}_{{\rm{s}}}{\hat{a}}_{{\rm{i}}}\right\rangle$, corresponding to three-fold coincidence events arising from multi-photon contributions of the signal photons. Since this term is not associated with the TPI, it contributes a background to the denominator of the visibility expression, leading to TPI visibility reduction.

As the system efficiency of the warm ^133^Cs ensemble decreases, loss of one photon from a signal-idler photon pair breaks the pairwise correlation. The surviving photon contributes to uncorrelated background, thereby reducing experimental TPI rate. By contrast, the high system efficiency of our warm ^133^Cs ensemble suppresses this uncorrelated background, enabling more robust TPI than the systems of lower efficiency at the same mean photon number ($\bar{n}$) conditions. High TPI visibility also requires low ${R}_{{\rm{s}}/{\rm{QD}}}$. In the QD system, high $\mu$ directly increases the single photon count rate, thereby lowering ${R}_{{\rm{s}}/{\rm{QD}}}$ and enhancing TPI visibility at fixed $\bar{n}$ of the warm ^133^Cs ensemble system. Therefore, to achieve high TPI visibility in experiments, it is desirable to improve the overall system efficiency and to reduce unwanted multi-photon events from the atomic ensemble.

Our result shows the first experimental TPI between two different types of quantum light sources without spectral modification of single photons from each source under CW operation. The current limitation in the TPI visibility arises primarily from two factors, the first one is the coherence time of single photons comparable to finite system response time. This can be addressed either by employing faster single-photon detectors or by extending the photon’s coherence time. In particular, the coherence time of single photons from the QD can be effectively extended through (quasi-)resonant excitation techniques^18^. Also, to avoid additional linewidth broadening during temperature-based frequency tuning^46,53^, applying an external field^54^ or strain tuning^55,56^ can expand the tuning range without reducing the coherence time. Second, to achieve near-unitary TPI visibility, ${R}_{{\rm{s}}/{\rm{QD}}}$ should be reduced while maintaining $\bar{n}$; this requires improving the system efficiency on both sides. In our experiment, the relatively lower system efficiency on the QD side than that of the ^133^Cs ensemble setup was limited by several factors, including collection (0.27), transmission (0.14), and detection efficiency (0.50). (Supplementary Note 6) To improve the efficiency of the QD platform, vertically symmetric QD devices, having the maximum collection efficiency of 0.5, can be transferred onto a highly reflective substrate, which redirects downward-emitted photons into the collection optics^57^. Together with the detectors and optical components with substantially higher efficiencies, overall single-photon count rate can be enhanced by more than one order of magnitude^16,58^. With the ideal photon indistinguishability (${I}_{{\rm{id}}}=1$) and a tenfold improvement in our experimental system efficiency for the QD ($\mu \approx 0.2$), which is comparable to the warm ^133^Cs ensemble system, the TPI visibility is expected to exceed $0.95$ by achieving much lower ${R}_{{\rm{s}}/{\rm{QD}}}$.

## Discussion

A quantum network requires interfacing stationary and photonic qubits, with all qubits across distributed quantum nodes operating at identical wavelengths. In this study, we have demonstrated TPI between two photons generated from a thin atomic vapor cell and a cavity-coupled QD. Compared to previous works where the atomic ensemble was used as dispersive medium for single photons from QDs, our approach demonstrates direct TPI between photons from atomic and QD single-photon sources. In contrast to other approaches that relied on spectral modification to compensate for the spectral dissimilarity between different sources, our platform exhibits intrinsic optical compatibility with matched wavelength, comparable linewidth, and high brightness between photons from two distinct single-photon sources. These features enable direct TPI without spectral modification and are essential for efficient interfacing and modular hybrid quantum networks. This result marks a significant advance in the development of hybrid quantum architectures based on heterogeneous quantum systems.

Furthermore, the narrow linewidths of both sources enable time-resolved TPI under CW operation. When the photon coherence time is sufficiently longer than the system temporal resolution, TPI can be achieved using time-resolved coincidence detection under CW excitation with idler heralding. In this regime, although photon arrival times are probabilistic, coincidence events can be identified through idler-photon heralding. Temporal gating is still necessary to define coincidence events, however, it can be implemented at the detection/post-processing stage^59–61^. CW operation combined with time-resolved detection is compatible with remote entanglement swapping protocol between distant quantum nodes based on continuous coincidence detection^62^.

Beyond the demonstration of TPI, our atom and QD hybrid system has several complementary strengths. QDs provide bright, on-demand single-photon generation and atomic vapor cells offer efficient absorbers of single photons, such that each can function as quantum light sources and memories. In another application of this hybrid system, atoms can provide a global frequency reference for ensuring spectral identity between remotely separated QDs^61^. When QDs are far separated, it becomes difficult to maintain their frequency matching, in this case, atomic resonances can provide local frequency standards, enabling independent stabilization of distant QDs and ensuring long-term spectral identity across distributed nodes.

The demonstrated TPI between these heterogeneous platforms, therefore, not only shows their optical compatibility, but also bridges the gap between photon generation and storage, and provides a global frequency standard for remote quantum emitters. In the future, extending this platform to Bell-state measurements will enable entanglement swapping and quantum teleportation^63,64^, providing a scalable and practical route to a modular hybrid quantum system and an essential step toward future quantum network architectures.

## Materials and methods

### InAs/GaAs sample design and fabrication

For the optimization and fabrication of the cavity, we followed our previous work^18^. The hole-based circular Bragg grating cavity was optimized for maximizing the collection efficiency of $907$ nm emission using a finite-difference time-domain method. Optimized parameters are radial direction periodicity: $\varLambda =322$ nm, center disk diameter: $R=2.6\varLambda$, tangential direction periodicity: $w=0.43\varLambda$, and hole diameter: $r=0.30\varLambda$. The optimized Gaussian far-field exhibits directional emission within 5-degree divergence.

Via the molecular beam epitaxy method, self-assembled InAs QDs embedded in 160 nm GaAs were grown. We deposited an additional Si_3_N_4_ layer with a thickness of 95 nm. We made a dry etch mask using electron beam lithography and dry etched Si_3_N_4_ and GaAs layers using reactive ion etching processes. To make the sample an air-suspended membrane structure, we selectively etched the AlGaAs sacrificial layer under the GaAs layer using diluted hydrofluoric acid.

### Experimental setup

In one arm, a 1-mm-long cesium vapor cell, heated to $105$ °C, was simultaneously illuminated by the CW $852$ nm pump and $917$ nm coupling lasers arranged in a counter-propagating geometry. The lasers were detuned by $+1.35$ GHz (one-photon) and $-40$ MHz (two-photon), respectively. While the coupling laser power was fixed at $15$ mW, the pump laser was varied below $0.03$ mW to adjust the heralded single-photon generation rate. The phase-matched photon pairs were emitted at specific angles; the signal and idler photons, emerging at approximately a $2$-degree angle with respect to the counter-propagating lasers, were filtered with a band-pass filter and polarizer to serve as the heralded single-photon source. Across all experimental settings, the heralding efficiency, defined as the ratio of coincidence counts to idler counts, remained above $9 \%$ ($18 \% ,\,22 \%$) within $80$ ps ($320$ ps, $2$ ns) experimental heralding time window.

In the other arm, the InAs QD was cooled down to $7.5$ and $12.4$ K using a closed-cycle He-flow cryostat (ST-500, Lake Shore) and excited by the CW laser of $780$ nm. Then, polarization-filtered QD emission was coupled into one input port of a fiber interferometer. Before the two-photon interference, the single photons were spectrally isolated using a grating-based fiber band-pass filter (WL Photonics) of $50$ GHz bandwidth.

At the $50:50$ beam splitter, the QD photon and the heralded signal photon were superposed, with PCs adjusted to parallel or orthogonal polarization to enable or suppress two-photon interference. Three-fold coincidence counts among the idler and two output channels of the interferometer were recorded to quantify the two-photon interference visibility. All photons were detected using SNSPDs optimized near $780$ nm, with quantum efficiencies of approximately $70 \%$ at $852$ nm and$\,50 \%$ at $917$ nm, and a timing jitter of ~$70$ ps FWHM. Time-tagging and correlation analysis were performed with a quTAG-MC (qutools) TCSPC, which provides $50$ ps FWHM timing resolution. For precise spectrum measurement, we used the scanning Fabry-Perot interferometer (SA210-8B, Thorlabs) with a free spectral range of $10$ GHz and a finesse of $160$, combined with a function generator to adjust the interferometer’s transmission frequency.

## Supplementary information

Supplementary Information

## Data Availability

The data that support the findings of this study are available from the corresponding authors upon request. The custom codes for simulating the quantum system are available from the corresponding authors upon request.
