# Supplementary material for: Two-photon interference between independent atomic and quantum dot single-photon sources for hybrid quantum network
Source: Light Sci Appl. 2026 Jul 15;15:320. doi: 10.1038/s41377-026-02399-y (PMC13373215; doi:10.1038/s41377-026-02399-y)
Supplement: Supplementary file 1 — Supplementary Information [file 41377_2026_2399_MOESM1_ESM.pdf]

Supplementary Information for

Two-photon interference between independent atomic and quantum dot single-photon sources for hybrid quantum network

Kyu-Young Kim[1†], Heewoo Kim[2†], Dong Hyun Park[1], Jinhyuk Bae[2], Gyeongmin Ju[2], Suk In Park[3], Jin Dong Song[3], Je-Hyung Kim[1]* and Han Seb Moon[2,4]*

[1]Department of Physics, Ulsan National Institute of Science and Technology; Ulsan, 44919, Republic of Korea

[2]Department of Physics, Pusan National University; Busan, 46241, Republic of Korea

[3]Center for Opto-Electronic Materials and Devices Research, Korea Institute of Science and Technology; Seoul, 02792, Republic of Korea

[4]Quantum Sensors Research Center, Pusan National University; Busan, 46241, Republic of Korea

[†]These authors contributed equally to this work.

[*]Corresponding authors. Emails: jehyungkim@unist.ac.kr and hsmoon@pusan.ac.kr

Supplementary Note 1. Correlation and coincidence measurements.

To measure the cross-correlation, $g_{i,s}^{(2)}(\tau)$, between idler and signal photons from the atomic ensemble, we prepared two detection channels, where Sync and Ch1 detected idler and signal photons, respectively. (Supplementary Figure 1a) We then recorded correlation between two channels, which corresponds to unnormalized second-order correlation. We normalized it by the accidental level, corresponding to the unnormalized second-order correlation at infinite time delay.

For the conditional second-order correlation, $g_{i,s,s}^{(2)}(\tau)$, we prepared three detection channels in which Sync detected idler photons, while Ch1 and Ch2 detected signal photons after passing through a beam splitter, as described in Supplementary Figure 1b. First, we measured the three-fold coincidence among three detectors. To obtain $g_{i,s,s}^{(2)}(\tau)$, we normalized the three-fold coincidence following [1].

In the case of Hong-Ou-Mandel (HOM) interference of heralded signal photons, Sync1 and Sync2 detected idler photons while Ch1 and Ch2 detected signal photons after two-photon interferences at a beam splitter, as described in Supplementary Figure 1c. We measured four-fold coincidences and normalized them by the accidental coincidence level at infinite time delay.

We prepared two detection channels to measure the second-order correlation of single photons from the QD, $g_{QD}^{(2)}(\tau)$, using two detection channels. Ch1 and Ch2 detected single photons after passing through a beam splitter as described in Supplementary Figure 1d, and we obtained the unnormalized second-order correlation. We normalized it by the accidental level, corresponding to the unnormalized second-order correlation at infinite time delay. The HOM interferences for the single photons were measured in the same configuration, except that the beam splitter was replaced with an asymmetric Mach-Zehnder interferometer. (Supplementary Figure 1e)

For the two-photon interference between single photons from the QD and the heralded signal photons from the atomic ensemble, three detection channels were used. Signal photons from the atomic ensemble and single photons from the QD were interfered at a beam splitter and detected by Ch1 and Ch2, while Sync detected idler photon, as described in Supplementary Figure 1f. We obtained three-fold coincidence. To quantify two-photon interference effect, we represent normalized three-fold coincidences in the manuscript by the accidental coincidence level at infinite time delay.

No background subtraction was applied to any of the data.

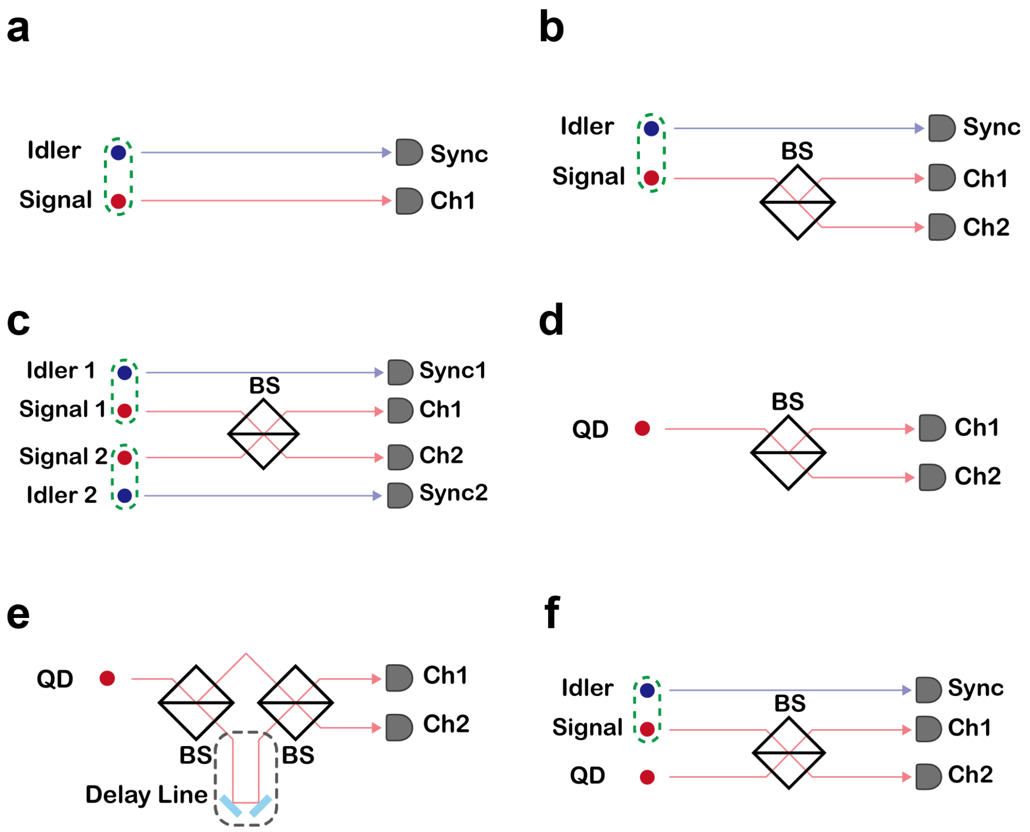

Supplementary Figure 1. Schematic of the experimental setup. Schematic of following experiments: **a**, The cross-correlation between signal and idler photons from the warm atomic ensemble ($\mathrm{g}_{\mathrm{i,s}}^{(2)}(\tau)$). **b**, The conditional second-order correlation of signal photons with idler heralding ($\mathrm{g}_{\mathrm{i,s,s}}^{(2)}(\tau)$). **c**, The Hong-Ou-Mandel interference of heralded signal photons ($\mathrm{g}_{\mathrm{HOM,i,i,s,s}}^{(2)}(\tau)$). **d**, The Hanbury Brown and Twiss experiment of single photons from the QD ($\mathrm{g}_{\mathrm{QD}}^{(2)}(\tau)$). **e**, The Hong-Ou-Mandel interference with unbalanced MZ interferometer ($\mathrm{g}_{\mathrm{HOM}}^{(2)}(\tau)$). **f**, The three-fold coincidence experiment between single photons from the QD and heralded signal photons from the warm atomic ensemble ($C_{\mathrm{i,s,QD}}^{\mathrm{dis}}(\tau)$).

Supplementary Note 2. Photon statistics of signal photons from ^{133}Cs with and without the heralding process.

Signal photons from the ^{133}Cs ensemble without heralding process using idler photons follow thermal distribution, which is $\mathrm{g}_{\mathrm{s,s}}^{(2)}(0) = 2$. To confirm the thermal property of the signal photons, we performed second-order measurement without any heralding process, as shown in Supplementary Figure 2a. Supplementary Figure 2b is the experimental result fitted with an equation of the same form as Eq. (3) of the main text. The bunching profile of $\mathrm{g}_{\mathrm{s,s}}^{(2)} = 1.90 \pm 0.03$ with a time scale of 418 ± 19 ps as a thermal light represents the well-known thermal distribution of the signal photons.

To suppress uncorrelated background photon detection, we apply a narrow 80 ps time window at the three-fold coincidence of heralded signal photons ($C_{\mathrm{i,s,s}}^{(2)}(\tau)$) using the idler photons as heralding triggers. (Supplementary Figure 2c) The heralding process effectively suppresses the probability of measuring uncorrelated signal photons and lowers the background level of uncorrelated signal photons ($\mathrm{g}_{\mathrm{s,s}}^{(2)}(\infty)$), which would otherwise exhibit thermal distribution in the absence of the heralding process. As a result, the zero-time delay bunching amplitude, normalized to the accidental coincidence level, increases to $C_{\mathrm{i,s,s}}^{(2)}(0) = 2.22 \pm 0.05$ (Supplementary Figure 2d) with a time scale of 263 ± 16 ps when we fit the data using an equation same form as Eq. (3) of the main text.

However, because of a large difference between the pronounced coincidence within the narrow time window around zero-time delay and the low constant background level, this three-fold coincidence ($C_{\mathrm{i,s,s}}^{(2)}(\tau)$) cannot exhibit the single-photon nature of heralded single photons and instead exhibit photon bunching. To reveal the anti-bunching profile of heralded single photons, we have to normalize the three-fold coincidence measurement result properly by the signal-idler cross-correlation (Inset of Fig. 3a of the main text) to compensate for the temporal photon distribution. As a result of normalization, we obtained the conditional second-order correlation (Supplementary Figure 2e or Fig. 3a of the manuscript) of the heralded signal photons with anti-bunching profile.

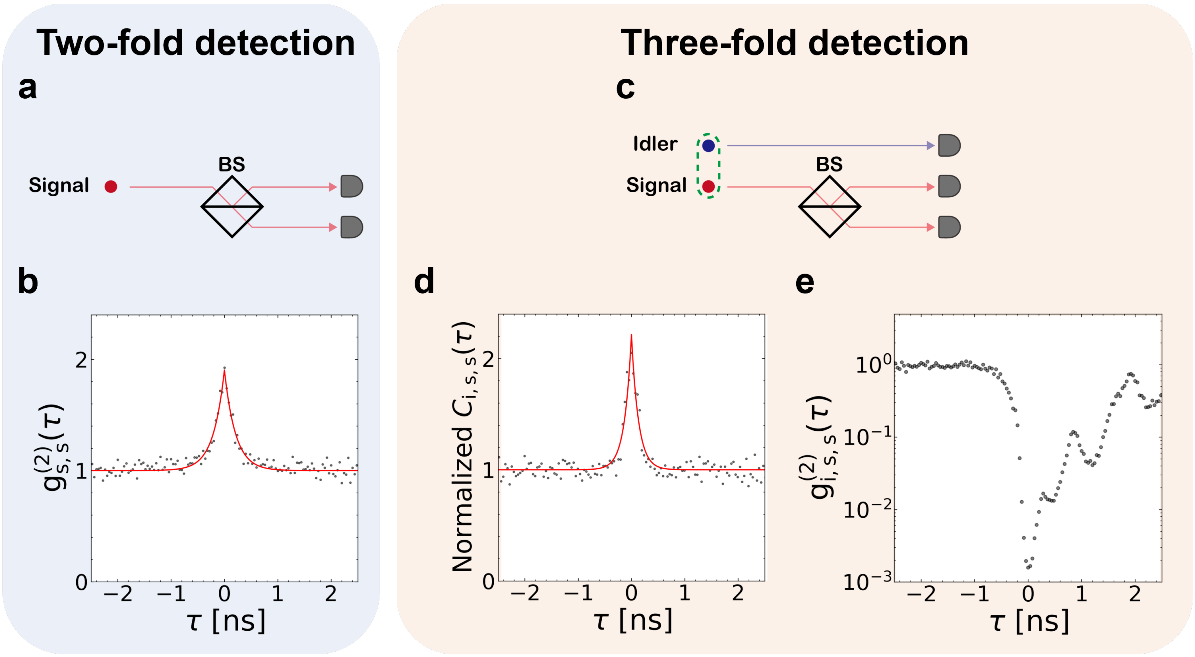

Supplementary Figure 2. Experimental schematics for correlation and coincidence measurements without and with heralding process and results. a, Schematic of the second-order correlation measurement of signal photons without the heralding process. **b**, Second-order correlation measurement result of signal photons without the heralding process. Because of thermal distribution, $g^{(2)}_{s,s}(0) \approx 2$. **c,** Schematic of three-fold coincidence measurement of signal photons with idler heralding trigger. **d**, Three-fold coincidence measurement result, normalized to the accidental coincidence level at the large time delay, of signal photons with heralding process using idler photons. **e,** Conditional second-order correlation of signal photons with heralding process after normalization by the cross-correlation between signal and idler photons. This data is the same as Fig. 3a of the manuscript.

Supplementary Note 3. Finite temporal resolution of the system and deconvolution process

All time-varying measurement systems have a finite temporal resolution. When this temporal resolution is comparable to or shorter than the timescale of the quantum dynamics of interest, the measurement results can be modified by the finite resolution of the measurement system. Therefore, it is necessary to account for the temporal resolution of the system and use appropriate equations to obtain accurate values and interpret the dynamics.

First, we quantify the temporal resolution of our system, consisted of SNSPD channels and TCSPC. The Gaussian FWHMs of the SNSPD channels are 63, 66, and 68 according to specification sheets, and the Gaussian FWHM of TCSPC is less than 50 ps. Then, the temporal resolution of the system can be defined as $\mathrm{FWHM}_{\mathrm{system}} = \sqrt{\sum_i \mathrm{FWHM}_{\mathrm{Ch}.i}^2 + \mathrm{FWHM}_{\mathrm{TCSPC}}^2}$, where $\mathrm{FWHM}_{\mathrm{Ch}.i}$ is the FWHM of the i'th SNSPD channel that we used. Therefore, the system resolution of 2- and 3-channels experiment is 104 and 124 ps. Then, we can reconstruct the Gaussian response function of our measurement system.

Next, we calculate modified fitting functions to analyze the dynamics. The slow system temporal resolution blurs measurement results and smooths out fast features. For example, the measured two-photon interference (TPI) dip becomes shallower than the actual one, and we underestimate the TPI visibility. To consider the finite system temporal resolution, we used a convolved fitting function with the Gaussian system response function. Let $f(\tau)$ is a fitting function, i.e. second-order correlation function, and $G(\mathrm{FWHM}_{\mathrm{system}}, \tau)$ be the Gaussian system response function with $\mathrm{FWHM}_{\mathrm{system}}$. Then, we numerically calculate the convoluted fitting function, $F(\tau) = \int dt\, f(t) \cdot G(\mathrm{FWHM}_{\mathrm{system}}, \tau - t)$ using the linear convolution method. From the fitting result, we can extract the unmodified results, so-called deconvolution process.

Because both distinguishable and indistinguishable datasets are presented within the same figures, showing all corresponding fitting curves would significantly increase complexity. We therefore display only the convolved fitting results in the manuscript and prepare additional figures including both convolved and deconvolved in this Supplementary note. Supplementary Figure 3 – 5 show measurement results with fitted results when the system resolution is finite (red solid line, $F(\tau)$) and ideal (blue dashed line, $f(\tau)$), respectively. In Supplementary Figure 3, the second-order correlation of single photons from the QD is deconvolved with 104 ps system temporal resolution. Supplementary Figure 4 show HOM result of single photons from QD when their polarizations are parallel, corresponding to the indistinguishable case, and the result of HOM visibility. In the fitting process, the system temporal resolution is 104 ps. The TPI between

signal photons from the warm Cs ensemble with heralding idler photons and single photons from the QD, and TPI visibility results are in Supplementary Figure 5. To correct the TPI visibility reduction caused by the finite system temporal resolution, we convolve a time-dependent TPI visibility function, as:

$$V_{\mathrm{i,s,QD}}(\tau) = V_{\mathrm{i,s,QD}}(0) \exp\left[-2|\tau|/\tau_{\mathrm{i,s,QD}}\right], \quad (1)$$

where $V_{\mathrm{i,s,QD}}(0)$ is the TPI visibility, and $\tau_{\mathrm{i,s,QD}}$ is the TPI coherence time, corresponding to the temporal overlap between the heralded signal photons from the warm Cs ensemble and single photons from the QD, with the system response function of 124 ps resolution, and the results are red (before correction) and blue (after correction) lines in Supplementary Figure 5.

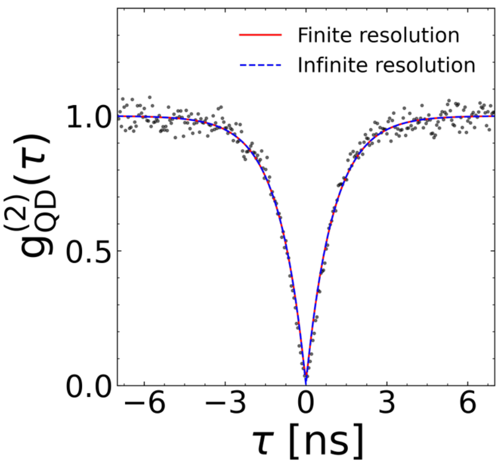

Supplementary Figure 3. Second-order correlation of single photons from the QD. Second-order correlation measurement result of single photons, which shows the single photon nature of $g^{(2)}_{\mathrm{QD}}(0) = 0.01 \pm 0.01$. Red solid and blue dashed lines are convolved and deconvolved fitting results.

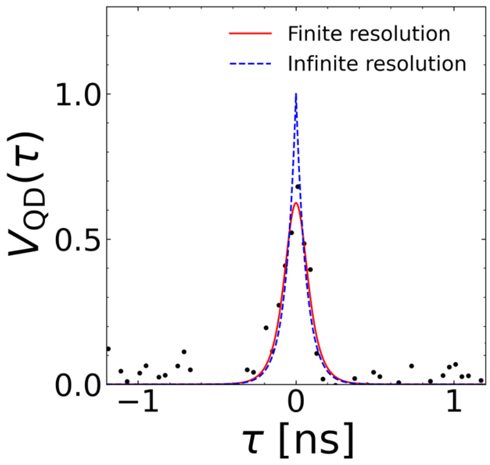

Supplementary Figure 4. HOM visibility result of single photons from the QD. HOM visibility result of single photons from the QD with deconvolution process when the sample temperature is 12.4 K. After correcting the finite system temporal resolution, the HOM visibility is 1 ± 0.10 with the coherence time of 129 ± 18 ps. Red solid and blue dashed lines are convolved and deconvolved fitting results.

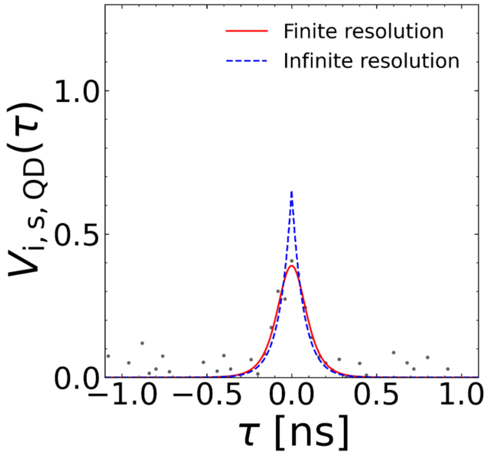

Supplementary Figure 5. TPI visibility between signal photons from the warm ^{133}Cs ensemble and single photons from the QD with the heralding process. TPI visibility result between signal photons from the warm ^{133}Cs and single photons from the QD with the heralding process when their unheralded intensity ratio is 0.25. Red solid and blue dashed lines are convolved and deconvolved fitting results.

Supplementary Note 4. The three-fold coincidence and the conditional second-order correlation.

In the manuscript, we introduce the conditional second-order correlation as follows [1];

$$g^{(2)}_{\mathrm{i,s,QD}}(\tau) = \frac{N_{\mathrm{i}}}{N_{\mathrm{i,1(2)}}} \cdot \frac{C_{\mathrm{i,s,QD}}(\tau)}{C_{\mathrm{i,2(1)}}(\tau)}, \tag{2}$$

where N_{i} and $N_{\mathrm{i,1(2)}}$ are the count rates of idler photons and the coincidences between heralding idler photons and the first (second) output port of the BS at zero-time delay, respectively, $C_{\mathrm{i,1(2)}}(\tau)$ is the two-fold coincidence between heralding idler photons and the first (second) output port of the BS, and $C_{\mathrm{i,s,QD}}(\tau)$ is the three-fold coincidence among idler and signal photons from the warm ^{133}Cs ensemble and single photons from the QD. Using this equation, the conditional second-order correlation at $R_{\mathrm{s/QD}} = 0.25$ in parallel polarization is Supplementary Figure 6.

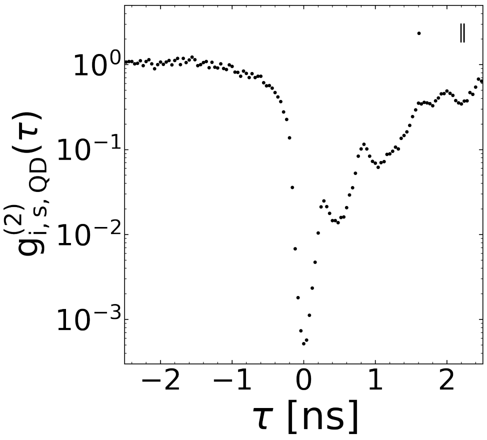

Supplementary Figure 6. The conditional second-order correlation of TPI. The conditional second-order correlation result of TPI between signal photons from the warm ^{133}Cs and single photons from the QD with the heralding process when their unheralded intensity ratio is 0.25 in parallel polarization.

Supplementary Note 5. Theoretical model

To analyze TPI between single photons and heralded single-photons, we calculated the third-order correlations. The single photons emitted from the QD are described as a Fock state $|\psi_{\mathrm{QD}}\rangle = \hat{a}_{\mathrm{QD}}^{\dagger}|0\rangle_{\mathrm{QD}} = |1\rangle_{\mathrm{QD}}$. In contrast, the quantum state of the heralded photon-pair generation from the warm ^{133}Cs ensemble is

$$|\psi_{\mathrm{s,i}}\rangle = \exp\left[r\left(\hat{a}_{\mathrm{s}}^{\dagger}\hat{a}_{\mathrm{i}}^{\dagger} - \hat{a}_{\mathrm{s}}\hat{a}_{\mathrm{i}}\right)\right]|0\rangle_{\mathrm{s}}|0\rangle_{\mathrm{i}} = \sqrt{1-\xi}\sum_{n=0}^{\infty}\xi^{\frac{n}{2}}|n\rangle_{\mathrm{s}}|n\rangle_{\mathrm{i}} \tag{3}$$

and

$$\xi = \tanh^2 r \tag{4}$$

where $\hat{a}_{\mathrm{s}}$ and $\hat{a}_{\mathrm{i}}$ are annihilation operators of signal and idler photon modes, and r is the degree of the squeezing. Under ideal condition of unitary system efficiency, including detection efficiencies of single photon detectors and transmission efficiencies of quantum channels, idler and signal photons are always co-existing and serve as single photons under heralding process, so-called heralded single photons.

Unfortunately, the real world is not ideal. The detection efficiencies of single photon detectors are less than 1, and there are always losses in quantum channels. The imperfect system degrades the intensities of quantum emitters, modifies quantum states of the quantum lights, and results in photons losing their pairs. To consider the lossy system, we adopt a beamsplitter approach. The lossy system can be expressed as a beamsplitter of which transmission efficiency is equal to the system efficiency. Then, the quantum states of photons after transmitting the beamsplitter is equivalent to the photons' quantum states at the lossy system. Let the system efficiency of single photons from the QD and signal(idler) photons from warm Cs ensemble be μ and $\eta_{\mathrm{s}}(\eta_{\mathrm{i}})$ which are less than 1. The unnormalized single photon state becomes

$$|\psi_{\mathrm{QD}}\rangle = |1\rangle_{\mathrm{QD}} \rightarrow |\psi'_{\mathrm{QD}}\rangle = \sqrt{\mu}|1\rangle \tag{5}$$

, and its intensity is $\mathrm{Tr}\left[\hat{a}_{\mathrm{QD}}^{\dagger}\hat{a}_{\mathrm{QD}}\right] = \mu < 1$. In contrast, the quantum state of the heralded photon-pair has multi-photon components. These multi-photon states are divided into several less-photon states with binomial distribution, like

$$|n\rangle \rightarrow \sum_{m=0}^{n}\sqrt{\binom{n}{m}}\eta^{\frac{m}{2}}|m\rangle. \tag{6}$$

Therefore, the unnormalized quantum state of the heralded photon-pair at the lossy system is

$$\begin{gathered}|\psi_{\mathrm{s,i}}\rangle = \sqrt{1-\xi}\sum_{n=0}^{\infty}\xi^{\frac{n}{2}}|n\rangle_{\mathrm{s}}|n\rangle_{\mathrm{i}} \\ \rightarrow |\psi'_{\mathrm{s,i}}\rangle = \sqrt{1-\xi}\sum_{n=0}^{\infty}\sum_{k=0}^{n}\sum_{l=0}^{n}\xi^{\frac{n}{2}}\sqrt{\binom{n}{k}\binom{n}{l}}\eta_{\mathrm{s}}^{\frac{k}{2}}(1-\eta_{\mathrm{s}})^{\frac{n-k}{2}}\eta_{\mathrm{i}}^{\frac{l}{2}}(1-\eta_{\mathrm{i}})^{\frac{n-l}{2}}|k\rangle_{\mathrm{s}}|l\rangle_{\mathrm{i}}\end{gathered} \tag{7}$$

and their intensity becomes

$$\mathrm{Tr}\left[\hat{a}_{\mathrm{s}}^{\dagger}\hat{a}_{\mathrm{s}}\right] = \eta_{\mathrm{s}}\bar{n} \tag{8}$$

and

$$\mathrm{Tr}\left[\hat{a}_{\mathrm{i}}^{\dagger}\hat{a}_{\mathrm{i}}\right] = \eta_{\mathrm{i}}\bar{n} \tag{9}$$

where $\bar{n} = \xi/(1-\xi)$ is the mean photon number of signal and idler photons. Therefore, count rate ratio between the signal and single photons is $R_{\mathrm{s/QD}} = \eta_{\mathrm{s}}\bar{n}/\mu$.

Compared to the previous TPI between two single photons [2,3], the TPI visibility between two photons with different statistics exhibits count rate ratio dependency. The single photons and the heralded signal photons meet at a 50:50 beamsplitter, and creation operators of $\hat{a}_{\mathrm{a}}^{\dagger}$ and $\hat{a}_{\mathrm{b}}^{\dagger}$ for input ports and $\hat{a}_{\mathrm{c}}^{\dagger}$ and $\hat{a}_{\mathrm{d}}^{\dagger}$ for output ports have relations that

$$\begin{pmatrix}\hat{a}_{\mathrm{c}}^{\dagger}\\ \hat{a}_{\mathrm{d}}^{\dagger}\end{pmatrix} = \frac{1}{\sqrt{2}}\begin{pmatrix}1 & i\\ i & 1\end{pmatrix}\begin{pmatrix}\hat{a}_{\mathrm{a}}^{\dagger}\\ \hat{a}_{\mathrm{b}}^{\dagger}\end{pmatrix}. \tag{10}$$

Successive TPIs occur only when the photons are indistinguishable, and the idler photons are heralded within the effective heralding time window. According to previous studies [1,4], analyzing conditional TPI requires third-order correlation for two output ports of the beamsplitter at time t_{c} and t_{d} and idler photons at time t_{i}, which is

$$C_{\mathrm{i,s,QD}}(t_{\mathrm{c}}, t_{\mathrm{d}}, t_{\mathrm{i}}) = \langle :\hat{a}_{\mathrm{i}}^{\dagger}(t_{\mathrm{i}})\hat{a}_{\mathrm{c}}^{\dagger}(t_{\mathrm{c}})\hat{a}_{\mathrm{d}}^{\dagger}(t_{\mathrm{d}})\hat{a}_{\mathrm{d}}(t_{\mathrm{d}})\hat{a}_{\mathrm{c}}(t_{\mathrm{c}})\hat{a}_{\mathrm{i}}(t_{\mathrm{i}}): \rangle \tag{11}$$

where $\hat{a}_{\mathrm{i}}^{\dagger}$ and $\hat{a}_{\mathrm{i}}$ are creation and annihilation operators for the idler photons. Using Supplementary Equation (10), the third order coincidence with output port operators at zero-time delay ($t_{\mathrm{c}} = t_{\mathrm{d}} = t_{\mathrm{i}}$) is defined as

$$C_{\mathrm{i,s,QD}}(0) = \left[\langle\hat{a}_{\mathrm{i}}^{\dagger}\hat{a}_{\mathrm{a}}^{\dagger}\hat{a}_{\mathrm{a}}^{\dagger}\hat{a}_{\mathrm{a}}\hat{a}_{\mathrm{a}}\hat{a}_{\mathrm{i}}\rangle + \langle\hat{a}_{\mathrm{i}}^{\dagger}\hat{a}_{\mathrm{b}}^{\dagger}\hat{a}_{\mathrm{b}}^{\dagger}\hat{a}_{\mathrm{b}}\hat{a}_{\mathrm{b}}\hat{a}_{\mathrm{i}}\rangle + 2\langle\hat{a}_{\mathrm{i}}^{\dagger}\hat{a}_{\mathrm{a}}^{\dagger}\hat{a}_{\mathrm{b}}^{\dagger}\hat{a}_{\mathrm{b}}\hat{a}_{\mathrm{a}}\hat{a}_{\mathrm{i}}\rangle(1 - I_{\mathrm{id}})\right] \tag{12}$$

where I_{id} is the degree of indistinguishability between the single and signal photons [5,6]. Hence, the conditional second-order correlation can be represented as

$$\mathrm{g}_{\mathrm{i,s,QD}}^{(2)}(0) = \frac{\langle\hat{a}_{\mathrm{i}}^{\dagger}\hat{a}_{\mathrm{i}}\rangle \cdot \langle\hat{a}_{\mathrm{i}}^{\dagger}\hat{a}_{\mathrm{c}}^{\dagger}\hat{a}_{\mathrm{d}}^{\dagger}\hat{a}_{\mathrm{d}}\hat{a}_{\mathrm{c}}\hat{a}_{\mathrm{i}}\rangle}{\langle\hat{a}_{\mathrm{i}}^{\dagger}\hat{a}_{\mathrm{c}}^{\dagger}\hat{a}_{\mathrm{c}}\hat{a}_{\mathrm{i}}\rangle \cdot \langle\hat{a}_{\mathrm{i}}^{\dagger}\hat{a}_{\mathrm{d}}^{\dagger}\hat{a}_{\mathrm{d}}\hat{a}_{\mathrm{i}}\rangle} = \frac{\langle\hat{a}_{\mathrm{i}}^{\dagger}\hat{a}_{\mathrm{i}}\rangle \cdot C_{\mathrm{i,s,QD}}(0)}{\left[\frac{1}{2}\left(\langle\hat{a}_{\mathrm{i}}^{\dagger}\hat{a}_{\mathrm{a}}^{\dagger}\hat{a}_{\mathrm{a}}\hat{a}_{\mathrm{i}}\rangle + \langle\hat{a}_{\mathrm{i}}^{\dagger}\hat{a}_{\mathrm{b}}^{\dagger}\hat{a}_{\mathrm{b}}\hat{a}_{\mathrm{i}}\rangle\right)\right]^{2}}, \tag{13}$$

of which the denominator is independent of I_{id}.

Therefore, the TPI visibility can be calculated through the following relation

$$V_{\mathrm{i,s,QD}} = \frac{\mathrm{g}_{\mathrm{i,s,QD},I_{\mathrm{id}}=0}^{(2)}(0) - \mathrm{g}_{\mathrm{i,s,QD},I_{\mathrm{id}}\neq 0}^{(2)}(0)}{\mathrm{g}_{\mathrm{i,s,QD},I_{\mathrm{id}}\neq 0}^{(2)}(0)} = \frac{C_{\mathrm{i,s,QD},I_{\mathrm{id}}=0}(0) - C_{\mathrm{i,s,QD},I_{\mathrm{id}}=1}(0)}{C_{\mathrm{i,s,QD},I_{\mathrm{id}}=0}(0)}. \tag{14}$$

Let's assume that the single and signal photons enter the input port a and b, respectively. Since the two photons are independent before TPI, the third order coincidence can be modified as

$$C_{\mathrm{i,s,QD}}(0) = \left[\langle\hat{a}_{\mathrm{i}}^{\dagger}\hat{a}_{\mathrm{i}}\rangle\langle\hat{a}_{\mathrm{a}}^{\dagger}\hat{a}_{\mathrm{a}}^{\dagger}\hat{a}_{\mathrm{a}}\hat{a}_{\mathrm{a}}\rangle + \langle\hat{a}_{\mathrm{i}}^{\dagger}\hat{a}_{\mathrm{b}}^{\dagger}\hat{a}_{\mathrm{b}}^{\dagger}\hat{a}_{\mathrm{b}}\hat{a}_{\mathrm{b}}\hat{a}_{\mathrm{i}}\rangle + 2\langle\hat{a}_{\mathrm{a}}^{\dagger}\hat{a}_{\mathrm{a}}\rangle\langle\hat{a}_{\mathrm{i}}^{\dagger}\hat{a}_{\mathrm{b}}^{\dagger}\hat{a}_{\mathrm{b}}\hat{a}_{\mathrm{i}}\rangle(1 - I_{\mathrm{id}})\right]. \tag{15}$$

The nth-order moments of the single and signal photons are

$$\begin{aligned} &\langle \hat{a}_{\mathrm{QD}}^{\dagger}\hat{a}_{\mathrm{QD}}^{\dagger}\hat{a}_{\mathrm{QD}}\hat{a}_{\mathrm{QD}}\rangle = 0, && \langle \hat{a}_{\mathrm{QD}}^{\dagger}\hat{a}_{\mathrm{QD}}\rangle = \mu \\ &\langle \hat{a}_{\mathrm{i}}^{\dagger}\hat{a}_{\mathrm{b}}^{\dagger}\hat{a}_{\mathrm{b}}^{\dagger}\hat{a}_{\mathrm{b}}\hat{a}_{\mathrm{b}}\hat{a}_{\mathrm{i}}\rangle = \mathrm{Tr}\left[\hat{a}_{\mathrm{i}}^{\dagger}\hat{a}_{\mathrm{s}}^{\dagger}\hat{a}_{\mathrm{s}}^{\dagger}\hat{a}_{\mathrm{s}}\hat{a}_{\mathrm{s}}\hat{a}_{\mathrm{i}}\hat{\rho}'_{\mathrm{s,i}}\right]/\mathrm{Tr}\left[\hat{\rho}'_{\mathrm{s,i}}\right] && \langle \hat{a}_{\mathrm{i}}^{\dagger}\hat{a}_{\mathrm{b}}^{\dagger}\hat{a}_{\mathrm{b}}\hat{a}_{\mathrm{i}}\rangle = \mathrm{Tr}\left[\hat{a}_{\mathrm{i}}^{\dagger}\hat{a}_{\mathrm{s}}^{\dagger}\hat{a}_{\mathrm{s}}\hat{a}_{\mathrm{i}}\hat{\rho}'_{\mathrm{s,i}}\right]/\mathrm{Tr}\left[\hat{\rho}'_{\mathrm{s,i}}\right] \end{aligned} \tag{16}$$

where $\hat{\rho}'_{\mathrm{s,i}} = |\psi'_{\mathrm{s,i}}\rangle\langle\psi'_{\mathrm{s,i}}|$ is the density matrix of the heralded photon-pair at the lossy system.

Supplementary Note 6. Efficiencies of the systems

In practice, system losses are significant and have the possibility to modify quantum states as we discussed above. Therefore, we separately evaluated efficiencies of the systems for QD and warm Cs ensemble. Supplementary Table 1 – 3 show optical components used in each system for the QD and warm Cs ensemble and their transmission efficiencies. As a result, the system efficiency for the single photons from the QD, μ, is 0.019, and the efficiency for the signal (idler) photons from the warm Cs ensemble, η_s (η_i), is 0.37 (0.57), respectively.

Optical components	Efficiency
Photon collection at the first objective lens	0.47
Objective lens (Mitutoyo / M Plan Apo NIR HR 100x)	0.70
Half wave plate (Thorlabs / AHWP05M-980)	0.99
Polarizing beamsplitter (Thorlabs / CCM1-PBS255/M)	0.97
Linear polarizer (Meadowlark Optics / UPM-050-Vis-AR2)	0.88
Mirror × 3 (Thorlabs / E03)	$(0.99)^3$
Half wave plate (Thorlabs / WPH05M-915)	0.90
Quarter wave plate (Thorlabs / WPQ05M-915)	0.90
Long pass edge filter (Thorlabs / FELH0900)	0.93
Objective lens (Newport / 5726-B-H)	0.84
Free space to fiber coupling	0.57
Grating-based fiber band pass filter (WL Photonics)	0.43
90:10 fiber beamsplitter (Thorlabs)	0.88
SNSPD (Quantum Opus)	0.50
**Total = μ**	**0.019**

Supplementary Table. 1. Listed optical components used for the single photons from the QD and their transmission efficiencies.

Optical components	Efficiency
Cell window	0.92
Linear polarizer (Meadowlark Optics / UPM-050-Vis-AR2)	0.88
Band pass filter (Thorlabs / FF01-920/10-25)	0.99
Fiber tip	0.96
Mirror × 2 (Thorlabs / E03)	$(0.99)^2$
Lens × 4	$(0.99)^4$
SNSPD (Quantum Opus)	0.50
**Total = $\boldsymbol{\eta}_\mathbf{s}$**	$\mathbf{0.37}$

Supplementary Table. 2. Listed optical components used for the signal photons from the warm Cs ensemble and their transmission efficiencies.

Optical components	Efficiency
Cell window	0.92
Linear polarizer (Newport / 05P109AR.16)	0.96
Band pass filter (Semrock / LL01-852-12.5)	0.99
Fiber tip	0.96
Mirror × 2 (Thorlabs / E03)	$(0.99)^2$
Lens × 4	$(0.99)^4$
SNSPD (Quantum Opus)	0.70
**Total = $\boldsymbol{\eta}_\mathbf{i}$**	$\mathbf{0.57}$

Supplementary Table. 3. Listed optical components used for the idler photons from the warm Cs ensemble and their transmission efficiencies.

References

1 Lee, Y.-S., Lee, S. M., Kim, H. & Moon, H. S. Highly bright photon-pair generation in Doppler-broadened ladder-type atomic system. *Optics Express* **24**, 28083-28091, doi:10.1364/OE.24.028083 (2016).

2 Kim, J.-H., Cai, T., Richardson, C. J. K., Leavitt, R. P. & Waks, E. Two-photon interference from a bright single-photon source at telecom wavelengths. *Optica* **3**, 577-584, doi:10.1364/OPTICA.3.000577 (2016).

3 Zhai, L. *et al.* Quantum interference of identical photons from remote GaAs quantum dots. *Nature Nanotechnology* **17**, 829-833, doi:10.1038/s41565-022-01131-2 (2022).

4 Höckel, D., Koch, L. & Benson, O. Direct measurement of heralded single-photon statistics from a parametric down-conversion source. *Physical Review A* **83**, 013802, doi:10.1103/PhysRevA.83.013802 (2011).

5 Wiegner, R., von Zanthier, J. & Agarwal, G. S. Quantum interference and non-locality of independent photons from disparate sources. *Journal of Physics B: Atomic, Molecular and Optical Physics* **44**, 055501, doi:10.1088/0953-4075/44/5/055501 (2011).

6 Khodadad Kashi, A., Caspani, L. & Kues, M. Spectral Hong-Ou-Mandel Effect between a Heralded Single-Photon State and a Thermal Field: Multiphoton Contamination and the Nonclassicality Threshold. *Physical Review Letters* **131**, 233601, doi:10.1103/PhysRevLett.131.233601 (2023).
